# When the aortoiliac bifucation is occluded:Leriche syndrome

**DOI:** 10.1016/j.amsu.2022.103413

**Published:** 2022-02-25

**Authors:** Said Adnor, Mehdi El Kourchi, Soukaina Wakrim

**Affiliations:** a Faculty of Medicine and Pharmacy, Ibn Zohr University of Agadir, Morocco; b Radiology Department, CHU Agadir, Morocco

**Keywords:** Leriche syndrome, Aortoiliac occlusive disease, CT angiography, Doppler ultrasound

## Abstract

**Intoduction:**

Leriche syndrome is a special type of obliterating arterial disease of the lower limbs which results in thrombotic occlusion of the aortoiliac junction.

**Case report:**

We report the case of a 65-year-old patient with known cardiovascular and nephrological pathological history, who presented with acute abdominal pain with intermittent claudication of the lower limbs and in whom clinical examination found abolition of the femoral pulses.

**Discussion:**

Doppler ultrasound of the abdominal aorta revealed aortic thrombosis in the lower of the renal segment extended to the iliac bifurcation with damping of upstream circulatory speeds. We supplemented with a CT angiography of the aorta and lower limbs which demonstrated extensive arterial thrombosis from the abdominal aorta to the bilateral external iliac arteries.

## Highlights

• Leriche syndrome is a special type of obliterating arterial disease of the lower limbs.
• It's Responsible of a unique triad of symptoms, including claudication, impotence, and decreased peripheral pulses.
• The diagnosis is suspected clinically, objectified by Doppler ultrasound and detailed by CT angiography.
• CT angiography is the examination of choice in the diagnosis of Leriche syndrome.

## 1 Introduction

Leriche syndrome involving an atheromatous occlusion of the infrarenal aorta, common iliac arteries, or both responsible of a unique triad of symptoms, including claudication, impotence, and decreased peripheral pulses [1,2]. It is a rare clinical entity [3] whose main risk factor is atherosclerosis [4].

The diagnosis is suspected clinically, objectified by Doppler ultrasound and detailed by CT angiography.

The purpose of this article is to shed light on this syndrome and the essential role of radiological examinations in the diagnosis.

## 2 Case report

A 65 years old patient, chronic smoker, hypertensive and chronic hemodialysis for 3 years, and having an hypokinetic heart disease, had presented for 1 month before admission low back pain with intermittent claudication and erectile disorders. To this symptomatology was added, 1 week before admission, excruciating abdominal pain.

Upon physical examination, we find a conscious patient (Glasgow score 15/15), normocardium (Heart rate 61 beats/min), normotensive artery, normopneic and the rightfemoral pulse is not perceived.

An aortoiliac doppler sonogram, and computed tomographic angiogram were ordered.

Ultrasound revealed aortic thrombosis localized in the lower third of the infra-renal segment, the lower end of which reaches the aortoiliac bifurcation, extended over 6.5 cm, stenosing, responsible for damping upstream circulatory speeds. While the iliac branches are permeable.

**Fig. 1:**
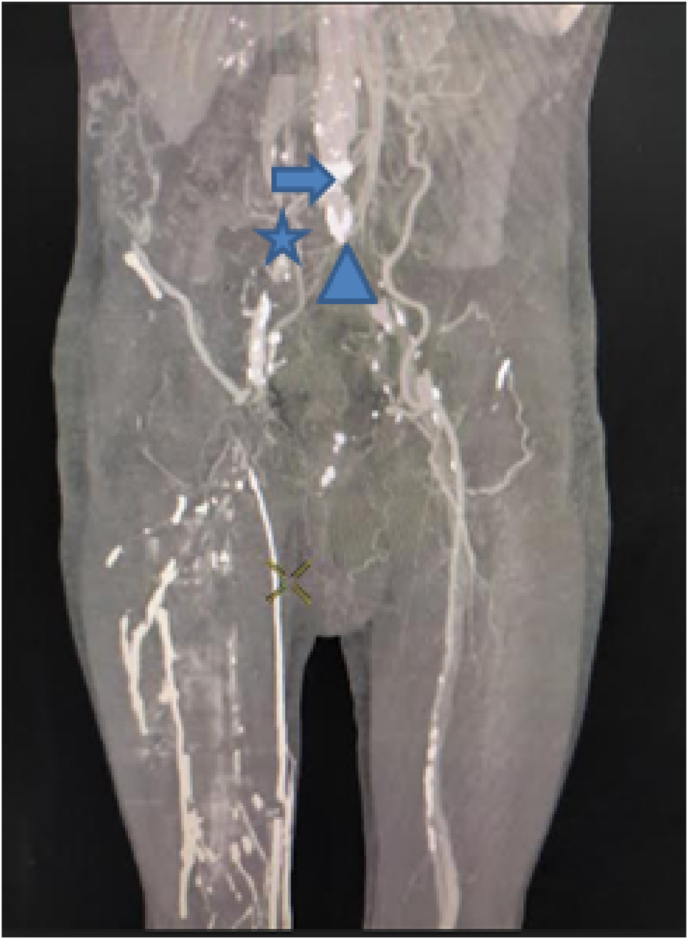
CT Angiography in coronal view showing a massive thrombus in the aortoiliac region Arrow: aorta star: right common iliac artery triangle: left common iliac artery.

**Fig. 2:**
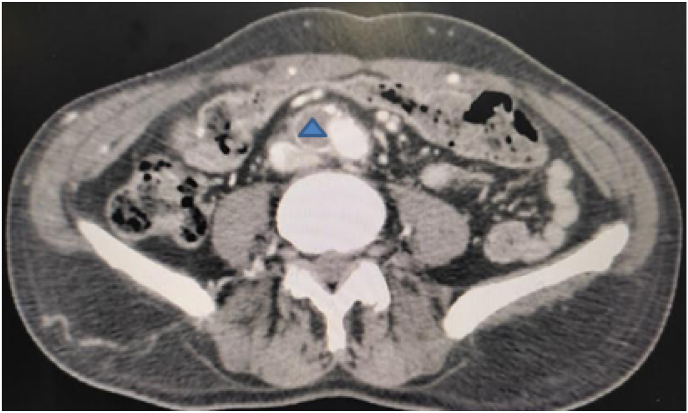
CT Angiography in axial view showing a massive thrombus in right common iliac artery (triangle).

**Fig. 3:**
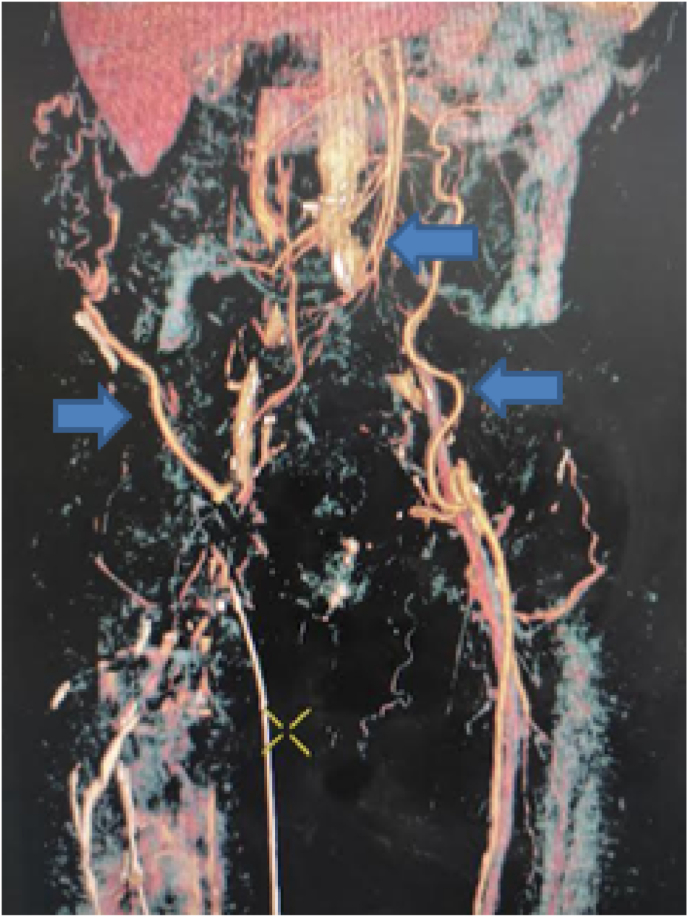
CT Angiography in coronal view volume rendering showing the presence of multiple collateral circulations (arrows).

The computed tomography (CT) angiography (Fig. 1, Fig. 2, Fig. 3) showed a massive thrombus at the aortoiliac bifurcation extending to the right common iliac artery, the right external and internal iliac arteries, and the left external iliac artery. The femoral arteries were permeable due to the presence of collateral circulations.

This work has been reported in line with the SCARE 2020 criteria [5].

## 3 Discussion

Leriche Syndrome (LS), also commonly referred to as aortoiliac occlusive disease (AIOD), is a product of atherosclerosis affecting the distal abdominal aorta, iliac arteries, and femoropopliteal vessels. LS was first described in 1914 by Robert Grahman, but it was not until later that the trio of symptoms was documented as a syndrome. This was done by Henri Leriche, a French surgeon, and physiologist, now known as the father of LS. When symptomatic, classically presents with a triad of claudication, impotence, and absence of femoral pulses. Claudication refers to cramping leg pain that is reproducible by exercise [6].

Leriche Syndrome is caused by atherosclerosis. Modifiable risk factors for atherosclerosis include hypertension, diabetes mellitus, nicotine, hyperlipidemia, hyperglycemia, and homocysteine. Non-modifiable risk factors for atherosclerosis include age, gender, race, and family history [4].

The average age of the patient at the time of diagnosis is around 50 years [7].

The lesions are generally limited to the aortoiliac level, the infrainguinal downstream bed is generally spared, the vital prognosis for the lower limbs is rarely involved and it is not a major atherosclerosis evolution [7].

The characteristic lesions of Leriche syndrome are of four types: a) isolated stenosis of the primary iliacs, b) more or less extensive lesions of the aortic bifurcation involving only the termination of the aorta and the origin of the primary iliacs, c) extensive lesions of the abdominal aorta and iliacs (as in our case), d) complete occlusion of the infra-renal aorta [7].

Doppler ultrasound is now a method of choice in diagnosis, evaluation and especially monitoring. It is non-invasive, inexpensive, reliable, reproducible but dependent on the operator, machine and patient [8].

Diagnostic imaging of Leriche syndrome and arterial obstruction of the lower limbs is generally based on CT angiography which is the most commonly used modality for the diagnostic and evaluation of patients with aortoiliac occlusive disease, allowing: a) excellent evaluation of stenotic arterial segments, b) determination if its total or partial occlusive nature, c) to assess its extent in particular to the primary iliacs, d) to determine the presence and importance of possible collateral circulation associated [7,9,10].

The treatment of Leriche syndrome is essentially surgical bypassing aorto-bi-iliac or aorto-femoral type, with low morbidity and mortality and excellent vascular permeability at a distance. Kissing transluminal angioplasty (with or without prior thrombolysis) is also becoming important as a first-line treatment, with good immediate results [7].

Complication: Limb ischemia [11] is a potential complication of LS as well as heart failure, myocardial ischemia/infarction, gangrene, and even death [12,13,14].

Prognosis: Without treatment, the prognosis of Leriche Syndrome is poor. However, with modern medicine outcomes are good. In some cases with slow progression or onset of LS, collaterals may develop as a self-compensating mechanism [15,16].

## 4 Conclusion

CT angiography is the examination of choice in the diagnosis of Leriche syndrome as it allows a high resolution vascular study of the aorta and arteries of the lower limbs and to determine the extent of the occlusion as well as the origin and extent of collateral circulation. This, with a view to planning the treatment in order to improve the functional prognosis of the patient.

## Provenance and peer review

Not commissioned, externally peer reviewed.

## Ethical approval

Written informed consent was obtained from the patient for publication of this case report and accompanying images. A copy of the written consent is available for review by the Editor-in-Chief of this journal on request.

## Please state any sources of funding for your research

The authors declared that this study has received no financial support.

## Author contribution

SAID ADNOR: Corresponding author writing the paper. SOUKAINA WAKRIM: Correction of the paper.

## Consent

Written informed consent was obtained from the patient for publication of this case report and accompanying images. A copy of the written consent is available for review by the Editor-in-Chief of this journal on request.

## Registration of research studies

1. Name of the registry:
2. Unique Identifying number or registration ID:
3. Hyperlink to your specific registration (must be publicly accessible and will be checked):

## Guarantor

SAID ADNOR.

## Declaration of competing interest

Authors of this article have no conflict or competing interests. All of the authors approved the final version of the manuscript.
